# Detection of occult non-mass enhancement in bloody nipple discharge using US/MRI fusion imaging: a case report

**DOI:** 10.1093/omcr/omag119

**Published:** 2026-07-08

**Authors:** Yukako Mouri, Masayuki Saito, Kanna Ozaki, Hirona Banno, Manami Goto, Mirai Ido, Takahito Ando, Junko Kousaka, Kimihito Fujii, Tsuneo Imai, Shogo Nakano, Takanori Ito, Emiko Takahashi, Koujiro Suzuki

**Affiliations:** Department of Breast and Endocrine Surgery, Aichi Medical University, 1-1 Yazakokarimata, Nagakute, Aichi 480-1195, Japan; Department of Breast and Endocrine Surgery, Aichi Medical University, 1-1 Yazakokarimata, Nagakute, Aichi 480-1195, Japan; Department of Breast and Endocrine Surgery, Aichi Medical University, 1-1 Yazakokarimata, Nagakute, Aichi 480-1195, Japan; Department of Breast and Endocrine Surgery, Aichi Medical University, 1-1 Yazakokarimata, Nagakute, Aichi 480-1195, Japan; Department of Breast and Endocrine Surgery, Aichi Medical University, 1-1 Yazakokarimata, Nagakute, Aichi 480-1195, Japan; Department of Breast and Endocrine Surgery, Aichi Medical University, 1-1 Yazakokarimata, Nagakute, Aichi 480-1195, Japan; Department of Breast and Endocrine Surgery, Aichi Medical University, 1-1 Yazakokarimata, Nagakute, Aichi 480-1195, Japan; Department of Breast and Endocrine Surgery, Aichi Medical University, 1-1 Yazakokarimata, Nagakute, Aichi 480-1195, Japan; Department of Breast and Endocrine Surgery, Aichi Medical University, 1-1 Yazakokarimata, Nagakute, Aichi 480-1195, Japan; Department of Breast and Endocrine Surgery, Aichi Medical University, 1-1 Yazakokarimata, Nagakute, Aichi 480-1195, Japan; Department of Breast and Endocrine Surgery, Aichi Medical University, 1-1 Yazakokarimata, Nagakute, Aichi 480-1195, Japan; Department of Surgical Pathology, Aichi Medical University, Japan; Department of Surgical Pathology, Aichi Medical University, Japan; Department of Radiology, Aichi Medical University, Japan

**Keywords:** bloody nipple discharge, non-mass enhancement, US/MRI fusion, ductal carcinoma in situ

## Abstract

A 66-year-old woman presented with right bloody nipple discharge (BND). Initial evaluation, including palpation, mammography (MMG), ultrasonography (US), and cytology, revealed no abnormalities. In the second year, persistent BND prompted breast magnetic resonance imaging (MRI), which demonstrated non-mass enhancement in the lower right breast and was classified as Breast Imaging Reporting and Data System (BI-RADS) category 3; therefore, imaging surveillance was performed. In the third year, cytological examination revealed atypical cells. Repeat MRI again demonstrated non-mass enhancement. US/MRI fusion identified two suspicious regions; however, ultrasound-guided needle biopsy showed no evidence of malignancy. Because of persistent BND and cytological atypia, excisional biopsy was performed, and ductal carcinoma in situ (DCIS) was confirmed. US/MRI fusion may assist in localizing target areas for biopsy in patients with MRI-detected lesions without corresponding findings on MMG or US; however, histopathological confirmation remains essential.

## Introduction

Bloody nipple discharge (BND) is a common clinical presentation of breast disease [1]. Further evaluation is often required when BND persists without corresponding findings on mammography (MMG) or ultrasonography (US). US/magnetic resonance imaging (MRI) fusion may assist in localizing target areas for biopsy in patients with MRI-detected lesions.

## Case report

A 66-year-old woman presented with right BND. Initial evaluation, including palpation, MMG (Fig. 1), US, and BND cytology, revealed no abnormalities.

**Figure 1:**
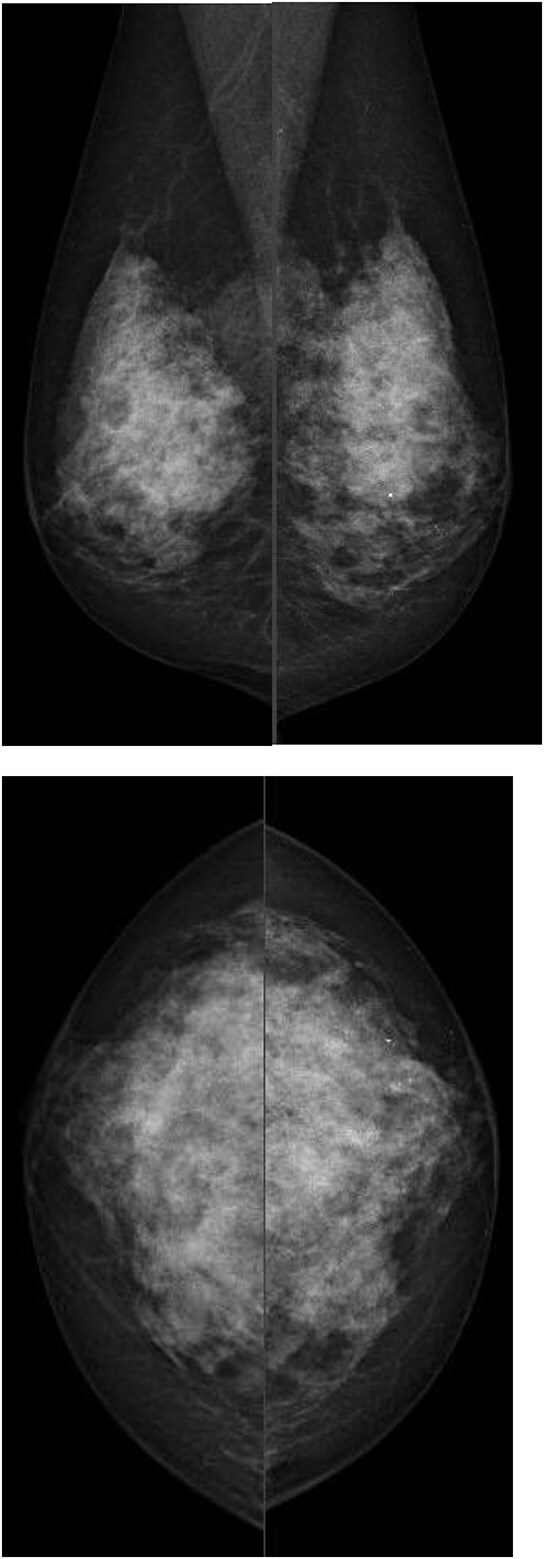
Mammographic findings. Mediolateral oblique (MLO) and craniocaudal (CC) views of the right breast demonstrated no abnormal findings. The examination was classified as category 1.

One year later, BND persisted. Repeat clinical examination and imaging remained unremarkable, and cytology was negative. Breast MRI (Fig. 2a and b) demonstrated diffuse non-mass enhancement (NME) in the lower right breast. The lesion was classified as Breast Imaging Reporting and Data System (BI-RADS) category 3 based on its diffuse distribution, heterogeneous internal enhancement pattern, and fast/wash-out kinetic features. As no corresponding lesion was identified on US, imaging surveillance was performed.

**Figure 2:**
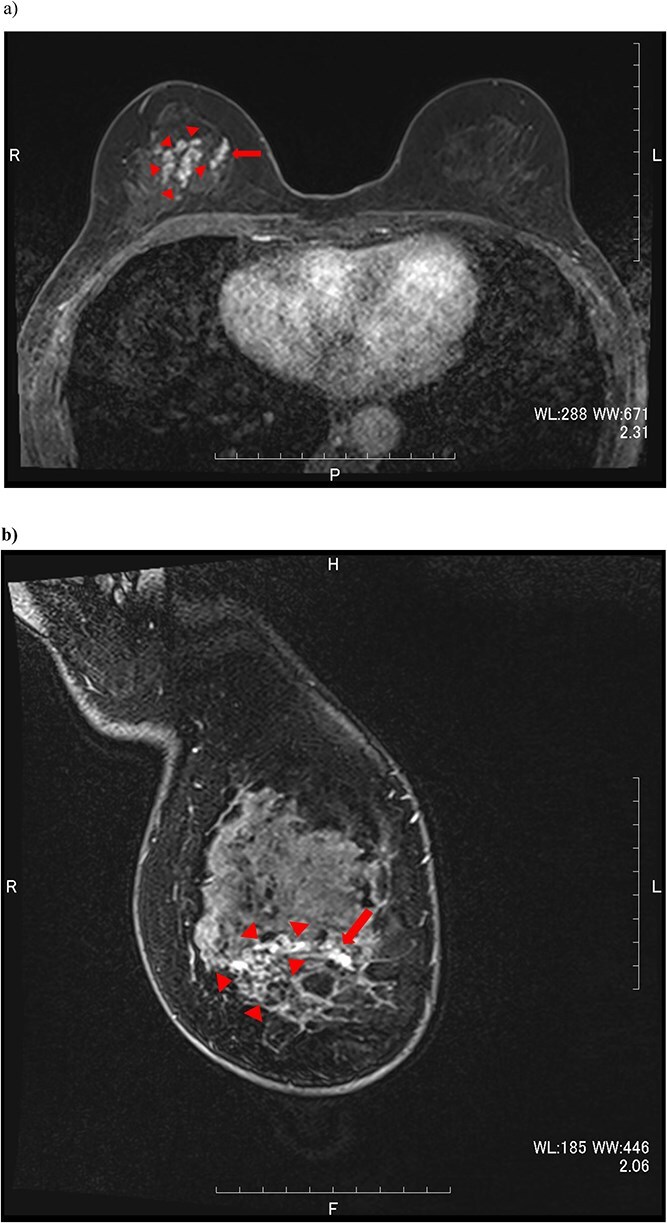
Breast magnetic resonance imaging findings. (a) Prone-position MRI. (b) Supine MRI. MRI demonstrated diffuse non-mass enhancement (NME) with heterogeneous internal enhancement in the lower region of the right breast. The lesion was assessed as BI-RADS category 3.

Intermittent BND recurred in the third year. Clinical examination and conventional imaging remained negative; however, cytology revealed atypical cells, although the sample was insufficient for a definitive diagnosis (Fig. 3a and b). Repeat MRI demonstrated findings similar to those of the previous examination. Because no correlating lesion was identified on US, US/MRI fusion was performed. Two suspicious regions were localized at the 3 and 9 o’clock positions (Fig. 4a and b). Ultrasound-guided needle biopsy of these regions showed no evidence of malignancy. Given the persistent BND and cytological atypia, wide excisional biopsy was performed. Histopathological examination revealed intermediate-grade solid and cribriform ductal carcinoma in situ (DCIS) without comedo necrosis in both specimens (Fig. 5a and b). Immunohistochemical analysis was not performed because the diagnosis was established based on routine histopathological findings.

**Figure 3:**
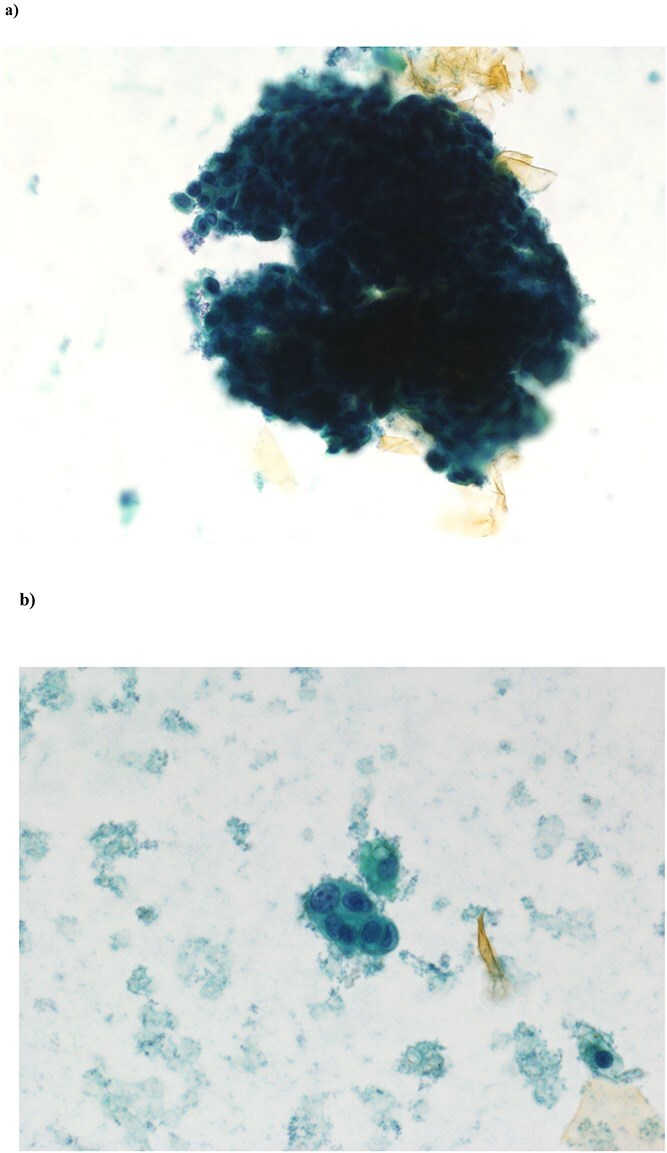
Cytological findings of bloody nipple discharge. (a) High-power view (×400) demonstrating a cluster of atypical ductal epithelial cells. (b) High-power view (×600) demonstrating atypical cells with nuclear pleomorphism and hyperchromatic nuclei.

**Figure 4:**
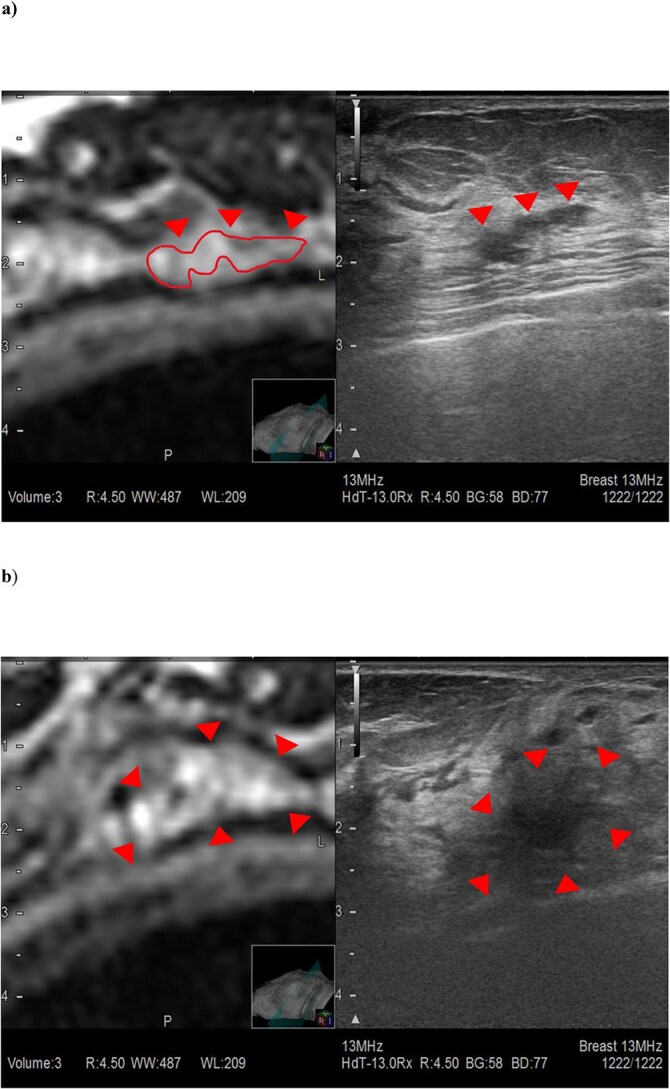
US/MRI fusion (real-time virtual sonography) findings. (a) US/MRI fusion localized a suspicious hypoechoic lesion in the 3 o'clock region measuring 9.0 × 4.6 mm. (b) US/MRI fusion localized a suspicious hypoechoic lesion in the 9 o'clock region measuring 12.5 × 6.6 mm.

**Figure 5:**
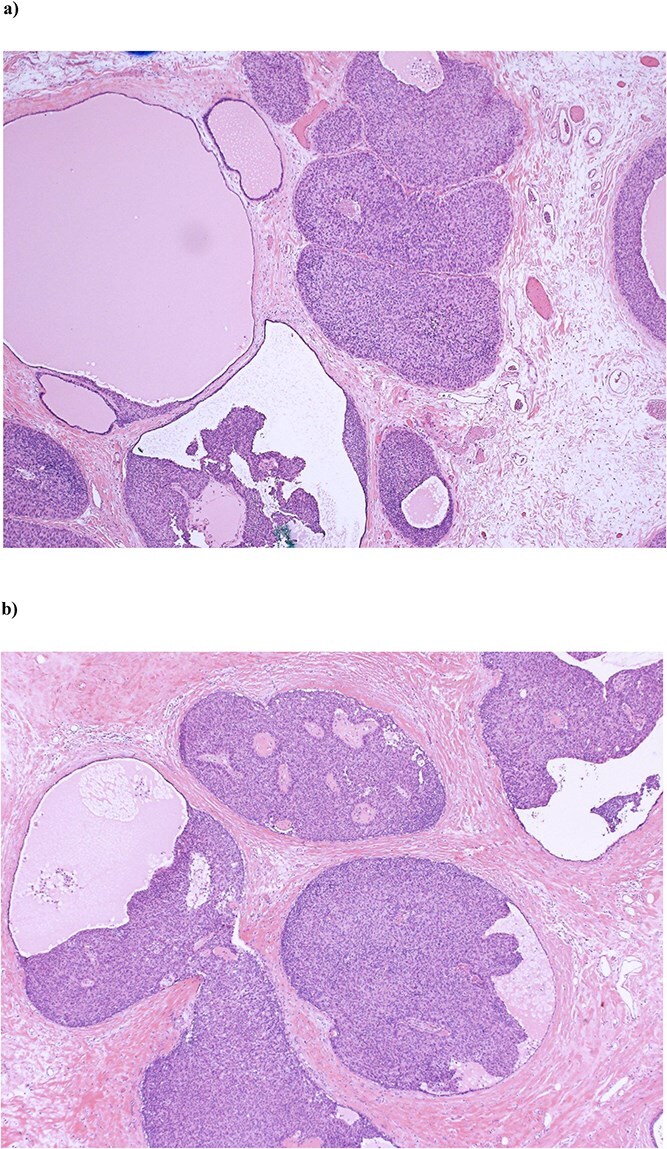
Histopathological findings of the excised specimens. (a) Low-power view (×40) of the specimen obtained from the 3 o'clock region. (b) Low-power view (×40) of the specimen obtained from the 9 o'clock region. Both specimens demonstrated similar histopathological findings. Moderately atypical ductal epithelial cells proliferated within dilated ducts, with occasional mitotic figures. The lesions were diagnosed as intermediate-grade solid and cribriform ductal carcinoma in situ (DCIS) without comedo necrosis.

The diffuse NME observed on MRI was considered to correspond pathologically to intraductal spread of DCIS. The clinical course is summarized in Table 1.

**Table 1:** Timeline of the patient’s clinical course and diagnostic findings.

BND	+	+	+	
MMG/US	-	-	-	
cytology	-	-	+atypical cell	
MRI	ND	+NME	+NME	
US/MRI fusion	ND	ND	+	

year				
	1	2	3	

BND: bloody nipple discharge.

- : normal finding + : abnormal finding.

ND: not done.

## Discussion

Nipple discharge (ND) is a common breast-related complaint and is classified as either physiological or pathological. Malignancy is identified in 3–23% of pathological cases, with unilateral BND being a concerning feature [1, 2]. Although most cases of DCIS are detected as calcifications on MMG, non-calcified DCIS can occur and may be missed [3, 4]. Such lesions may present as ductal abnormalities, complex masses, or architectural distortion [5].

MRI plays an important role in the detection of non-calcified DCIS. NME, particularly in a linear or segmental distribution, is a typical finding, whereas diffuse patterns, as observed in this case, are less common [5]. However, distinguishing benign from malignant NME remains challenging, and MRI findings alone are not always specific [6].

In cases in which BND is the only symptom and no correlates are identified on MMG or US, MRI findings may guide further evaluation. US/MRI fusion (real-time virtual sonography [RVS]) enables spatial correlation between MRI and real-time US and may assist in localizing target areas for biopsy. Previous studies have reported that second-look US using RVS can detect a substantial proportion of MRI-detected NME lesions, although not all lesions are identified [7].

In the present case, RVS enabled localization of two suspicious regions corresponding to MRI findings; however, needle biopsy did not reveal malignancy. The diffuse NME observed on MRI was considered to correspond pathologically to intraductal spread of DCIS. The final diagnosis of DCIS was established only after excisional biopsy because of persistent BND and cytological atypia. These findings indicate that RVS may be helpful for lesion localization but cannot replace histopathological confirmation.

Persistent pathological nipple discharge with cytological atypia may warrant surgical excision even when minimally invasive biopsy findings are negative.

This study has several limitations. First, this was a single-case report, and the findings may not be generalizable. Second, although lesion localization was achieved, the initial needle biopsy yielded negative results, highlighting the potential for sampling error. Third, supine MRI may be affected by respiratory and cardiac motion, potentially reducing image quality, and dedicated breast coils for supine imaging are not widely available. These factors may affect diagnostic accuracy in some cases. Therefore, careful clinical judgment is required when imaging and biopsy findings are discordant.

## Conclusion

US/MRI fusion may assist in localizing target areas for biopsy in patients with MRI-detected lesions without corresponding findings on MMG or US; however, histopathological confirmation remains essential.
